# Dental Hints

**Published:** 1867-12

**Authors:** A. B. Robbins


### DENTAL HINTS.

BY A. B. ROBBINS, D. D. S.

An Essay read before the Lake Erie Dental Association.

Thinking that a few hints might be of more use in promoting thought and directing some one to a higher style of practice, than a more elaborate essay on some specific subject would be, I have thrown together a few words at the risk of not doing justice to any one subject. Our practice, as well as our lives, are so largely made up of separate or individual action, that we each excel in some particular thing, just as we do that particular something, better than another.

Although I have a painful sense of my own ignorance and imperfections, yet with a ready willingness to learn, I hope to say something that shall at least strengthen the resolution of some one to attain a greater knowledge of our specialty, and by your discussions, be instructed myself.

But while I give prominence to trifles, let us consider some facts about these trifles.

The grandest architectural structures in the world are made up of small blocks of various hues and shapes. There is a *method* in the shaping of these blocks, each one of which is a small thing in itself, a careful blending of the colors, and not least, a beautiful symmetry of the whole.

Beneath the mass, and essential to its very existence, lies the deep foundation, firm and solid as the living rock. The finished pile ministers to the practical and æsthetic wants of man. It is a blessing to all—a thing of beauty and a joy forever The education of an accomplished Dentist is made up of comparatively trifling items. The foundation, a good general education, is laid deep and firm, and above it rises, inch by inch the superstructure, whose fair exterior shows harmonious combinations of many forms and hues. Finally, we hail as gentleman and scholar, skillful mechanic, scientific man and artist—the Dentist!

If we shall see, even now, the weak points and faulty places in our foundation, let us take Chicago skill to our aid, put the jackscrews to the work, and hold ourselves as well as we may, until we dig deep, vigorously pile in and secure a substantial resting place for our faith and practice. What I most fear is that the *even now* I speak of, may not appear to us, and we be made conscious of our weakness and need of aid. If we can only see our need, the first great advance has already been made, and I have no doubt of the result. The trouble is to get upon the stand point that will enable us to perceive that there is a field of beauty open to the vision of such as can see. We are daily walking over and in the midst of wondrous facts that are rapidly and rapturously unfolding to the eye of the scientific man, to which others are as blind as bats to the splendor of sunlight. We must endure home-thrusts and conscious probing. When our works are brought to the focal point and the strong light of truth let in upon them, imperfections stare us in the face. What then is our duty? Is it to go on with such poor results? Shall we not rather strive for higher attainments?

Then what is a good foundation, and what the sure stepping stones to a proper stand point? We all know that the mariner in seareh of land, leaves the hold of the ship and goes up, not only upon the deck, but even to the top of the mainmast, and then brings to his aid the tried and trusted spyglass. Let us follow his example, and from as elevated a position, take a general view of our surroundings.

The true, sure foundation of the Dental Art and Science is *Anatomy*, well laid upon the substratum of a sound general education. To such a beginning we can add *Physiology*, *Chemistry*, *Therapeutics*, *Pathology*, *Microscopy*, and *Mechanical Art*. To build up such a structure will be very hard for those who have been constructing of the fragments and spawls; harder still for those who have supposed themselves masters, and doing the labor of competent conservators; but let no one rest for a moment from the task of correct building, no matter how low he may have to dig, or how much rubbish remove.

It seems sometimes as if the public appreciation of what we ought to be, was higher than that of many in the profession. That such, however, is not universally the case, we have daily evidence. In a neighboring town, I once saw quite a display of Dental instruments on the parlor table of the hotel. I asked the Dental tyro whether he had many patients. "Oh," said he, "a very good number, *for the fools are not all dead yet.*"

So I often think when I see how many, ignorant of the first principles of our profession, succeed in obtaining a living. I would that we might soon see every one worthy of a good living. And now, with our Dental Colleges in every direction, most Dentists *may* be properly educated and fitted for their calling.

Let us then be up and doing;
With the heart and head begin;
Still achieving, still pursuing,
Learn to labor and to win!

If our birth denies us wealth,
Lofty state and power,
Honest fame and hardy health
Are a better dower.

Then let not an hour's delay
Cheat us of our due,
But, while it's called to-day,
Study, and our work pursue.

I wish to call your attention to my ever recurring theme, *the superiority of the natural teeth.* Some may be ready to exclaim, "Who doubts the superiority of the natural teeth?" I answer, I presume no one; and yet, I ask, do you always prove your faith by your works?" I cannot and need not picture to you the full beauty and uses of a well formed, sound and complete natural *denture;* and yet I wish such a picture were before us, so that not our ideal, but real appreciation of the beauty, strength, position, usefulness and value of a natural tooth might be ever present with us; that we could not *remove* or *mutilate* a tooth without realizing the destruction we have wrought, the injury and evils entailed. Think you that if the people and the Dentists placed a proper estimate on the teeth, there would be such a suicidal neglect of them? Would we ever hear it said, "I will let my teeth go and after a time have them all taken out, and get a new set!" A remark like this always sends a pang to my heart! I think of the lost beauty, the changed features, the crippled functions of the primæ viæ, the pallor, in a word, the misery and shortened days of such an one.

Now, my appreciators of natural teeth, do you put forth all your efforts to save the teeth that come under your care and treatment? Do you begin with the children when you can, and save the first or temporary teeth, and thus, so far as in your power, secure the correct dentition of the second or permanent teeth? When the permanent teeth are decayed upon their approximal surfaces, do you fully supply the lost portion with gold or other material, and fully restore the contour? How many still mutilate the molars so that they are no longer grinders, but make them *saw teeth* without setting them in a proper frame for sawing! So too, the other teeth we see daily cut and carved out of their natural shape and beauty.

You will pardon me for making some of my remarks emphatic by using personal pronouns. I do not intend to be personal. I only wish to *hint* at some of the mistakes made in our profession, and am fortunate if I may point out a more excellent way.

I do earnestly protest against the *unnecessary Extraction of teeth.* I do hope that not one of our members will try to improve on nature by *changing the shape of the teeth*, but when it is necessary to remove any considerable surface, will restore the tooth to its natural shape. Do not fear contour fillings; but do not cut teeth on purpose to put in such fillings, especially in the incisors for show. While I like to see teeth saved that have been plucked as brands from the burning, the frail darkened portions removed, and the edges carefully and nicely beveled, the tooth bleached if need be, and then like a precious gem, set in gold, I much prefer to have dental skill called in at an earlier period, before such sad havoc and ruin have become masters of the situation; when the edges and faces of the teeth are intact, so that when the gold has taken the place of removed dental tissue, the *gold* shall be as a precious gem set in dentine, and not the dentine set in gold.

To make myself fully understood, even at the risk of wearying you, I will in a measure restate the case.

The Dentist should first know the constitution, constituents, contents and connections of the teeth, and the contaminations that surround them.

2dly. The pathological condition and the remedy needed.

3dly. He should have the skill and ability to apply that remedy, so that the healed and restored teeth shall be as nearly as possible in their normal condition. I can never regard teeth that have been filed and chiseled to make room, and left with slots and V's, as normal.

Some use the file too much, others not enough. Some fill too full and keep the teeth forced into *new positions*. Others fill too flat and never restore the contour of badly decayed teeth. Some have never learned to articulate crown fillings. When these fillings are too full and the antagonizing teeth strike hard upon them, soreness, and often pain follow.

I know some good Dentists who wedge too much, and insert too many wedges at once; on this point *I can speak feelingly*. Others have never learned the advantage of the wedge. There is a happy medium to which I hope all may attain, and that is the great object of our investigations.

I am compelled only to glance at these important matters, and, as I said, only throw out hints. There is one point, small in itself, but valuable I think, that I will mention. I have spoken of it before elsewhere, but as there may be some who hear me, that have not tried it, I will describe it. It is the use of a shield or guard to prevent the abrasion of the edges of teeth, when removing decay, or correctly shaping approximal cavities; it is especially valuable when the drill is used, the shaft resting against thin enamel. The shield may be made of any available material; paper, tape, sheet rubber, or thin gold plate. I prefer the latter in most places. It is simply bent so as to pass between the teeth, and lap over the face of the tooth with a projecting strip or short handle to be used in retaining it in place while rotating the drill in the adjoining tooth.

You will often see little notches in the edges of the enamel made by the instrument, which the guard would have prevented. It is very important to preserve the outer coating and polish of enamel. When the opening or space between the teeth is small and cannot easily be enlarged, the thin sheet rubber, or a small ring cut from a piece of rubber tubing, will fully protect the tooth with ordinary care. Permit another hint, if you please. Some Dentists, since the subject of retaining points in cavities for the retention of fillings, has been brought to their notice, have simply drilled small pits in shallow or saucer shaped cavities and trusted to them, instead of making a good shaped cavity, which the condition of the tooth would permit. If you use sharp well tempered cutters, and square edged drills, and then, when circumstances will permit, cut so as to have well defined walls, that you feel and know, will retain well impacted gold, you will have confidence in your operations. When retaining points are needed, cut grooves if possible; but here let me caution against cracking off edges and periling your operation. In all cases, when you can without injury to the tooth, make your cavity *retentive*, without grooves or points.

We often meet with teeth that are soft, fibrous, rapidly dissolving, and present genuine specimens of white decay. When cut, they seem rather cartilaginous than osseous; the enamel friable, frequently pearly, sometimes chalky. To properly prepare such teeth for filling and secure hard solid surroundings, would often expose the pulp and destroy the vitality of the teeth. Let me mention a case in point:

A young lady, twenty years of age, called to have her teeth filled. I found them very soft and sensitive; on attempting to remove the decayed portion, I found the dentine almost destitute of solids. I decided not to operate, and commenced treating her to secure the infiltration of lime salts; to my surprise, in two weeks I found the same dentine hardening, even giving off the mineral ring: in two or three weeks more, her general health was much improved, and her teeth were well hardened, or as some would say, calcified. I filled them and feel confidence in the result.

I wished to speak of the result of treatment of the mother, to secure good teeth in the child, and the necessary steps to secure future development and preservation. I cannot at this time, but hope to do so at some future period.

Active professional duties and the state of my health, have prevented me from fulfilling my intentions, and leave to you only these fragments.

